# Medical Information on the Internet: A Tool for Measuring Consumer Perception of Quality Aspects

**DOI:** 10.2196/ijmr.3144

**Published:** 2015-03-30

**Authors:** Arthur Dubowicz, Peter J Schulz

**Affiliations:** ^1^Institute of Communication and HealthUniversity of LuganoLuganoSwitzerland; ^2^Department of Society and Human DevelopmentHarvard School of Public HealthBoston, MAUnited States

**Keywords:** online health information, scale development, quality assessment, sleeping disorders, Internet intervention

## Abstract

**Background:**

Most of adult Internet users have searched for health information on the Internet. The Internet has become one of the most important sources for health information and treatment advice. In most cases, the information found is not verified with a medical doctor, but judged by the “online-diagnosers” independently. Facing this situation, public health authorities raise concern over the quality of medical information laypersons can find on the Internet.

**Objective:**

The objective of the study was aimed at developing a measure to evaluate the credibility of websites that offer medical advice and information. The measure was tested in a quasi-experimental study on two sleeping-disorder websites of different quality.

**Methods:**

There were 45 survey items for rating the credibility of websites that were tested in a quasi-experimental study with a random assignment of 454 participants to either a high- or a low-quality website exposure. Using principal component analysis, the original items were reduced to 13 and sorted into the factors: trustworthiness, textual deficits of the content, interferences (external links on the Web site), and advertisements. The first two factors focus more on the provided content itself, while the other two describe the embedding of the content into the website. The 45 survey items had been designed previously using exploratory observations and literature research.

**Results:**

The final scale showed adequate power and reliability for all factors. The loadings of the principal component analysis ranged satisfactorily (.644 to .854). Significant differences at *P*<.001 were found between the low- and high-quality groups. Advertisements on the website were rated as disturbing in both experimental conditions, meaning that they do not differentiate between good and bad information.

**Conclusions:**

The scale reliably distinguished high- and low-quality of medical advice given on websites.

##  Introduction

### Health Information and the Internet

Internet usage is increasing strongly as more and more people have access to it. The increase reaches all age groups, including older people [1,2]. As a result, the amount and the use of health-related information on the Internet are also growing. Several studies show that, for health information, the Internet is one of the primary resources [3-7]. The Internet has thus become one of the most important sources for health information and for searching health care services and treatment advice. Data show that, within a given year, about 80% of adult Internet users have searched for health information [3,8]. In Germany in 2007, 56.6% of Internet users described their use as health-related [9]. In comparison to a previous study, Germany was among the European countries with the highest growth in this segment [9]. The age group searching most actively for health information was young adults between the ages of 30 and 44 years [10]. Data also show that, with a higher usage and availability of the Internet in general, Internet health usage grew across all age groups and among both genders [9]. About 35% of people searching for health information use the information they find to diagnose their medical condition. Only half of these so-called “online-diagnosers” check their diagnosis with a medical professional [3].

Cost and time factors make searching the Internet an attractive alternative to seeing a doctor in a nonacute situation, as information is available immediately and a visit to one’s doctor can be (work) “time consuming”. Individual reasons for searching medical information might differ—some want to prepare for a medical doctoral consultation, others seek support, or alternative remedies to treatment advice—but the accuracy of search results is significant for “online-diagnosers”. Hence, public health authorities are concerned over the quality of the health information available on the Internet [10]. A review on mental disorder information websites came to the conclusion that most scholarly articles report poor quality [11]. Erroneous, misleading, or irrelevant health information provided on the Internet can lead to wrong self-diagnosis and ineffectual or damaging treatment attempts by the layperson, and to delayed presentation at a general practitioner or hospital, which in turn can make therapy more difficult. This risk is especially increased by the fact that most of the information found on the Internet is not discussed with a medical practitioner, but rather used as the single basis for making a decision [8,12]. In addition, information acquired from the Web might make patients less willing to adhere to their doctor’s advice, and thus result in poor health outcomes. Finally, there is also the possibility of financial damages if a patient decides, based on bad advice from websites, to buy over-the-counter medication or equipment that does not provide remedy. Health-related decisions of individuals can be understood as affected by health literacy, which is the ability to understand medical information and to pass adequate judgment in matters of health [13,14]. The ability to distinguish good advice from bad advice can therefore be considered an aspect of health literacy.

### Sleeping Disorders

A very common medical condition in the general population is sleeping disorders or insomnia. About 50% of the population complains about such problems in a given year, and it is the most common complaint of patients after general pain [15]. Moreover, most people suffer from sleeping disorders periodically, and often have to rely on self-treatment when not at a doctors [16-18]. A lot of information on this condition can be found on the Internet. This material is very diverse, and the corpus consists of medical information, individual reports, advertisements, as well as alternative remedies. Moreover, producers of over-the-counter sleeping medication advertise their products heavily. As there is so much and such diverse information, its quality becomes difficult to judge. Additional research has shown that the accuracy of health information depends on the topic; information on more specific diseases is of higher quality than information on general health problems [19]. Especially in this context, sleeping disorders can be seen as a condition with much low-quality information. For this study, the whole range of sleeping disorders was incorporated, and no selection was applied as to whether it was a primary disease or a symptom.

### Credibility of Internet Health Information

The understanding of trust and credibility factors of Internet health information, and websites in general, has been addressed by research in recent years. Accordingly, various measures and quality criteria for health information on the Internet can be found [19-28]. An often-found approach is based on expert or consumer ratings of health information [25,29-31]. The DISCERN scale and its adaptation for the eHealth context are the prime examples, assessing health information quality with regard to patients’ treatment decisions [32,33]. In contrast, our measure tries to take the particular setting of Internet information into consideration. The DISCERN scale was developed for health or communication professionals and experienced users who want to discriminate between high- and low-quality health information. In contrast, our approach tried to take the particular setting of Internet health information into consideration and puts the average user of health information into its focus [33]. Information usage on the Internet is characterized by the short attention given by the consumers and a comparison of different sources [3,5,7].

A recent review described some of the tools for assessing the quality as having limited validity [11]. Still, most of these tools lack empirical testing and provide mostly conceptual work [28]. Reviews in the field mention the lack of an overall framework to assess this domain, and the need for a feasible definition of quality criteria for the websites [19]. There is also research on the process of how consumers assess medical information on websites [34]. Another line of research is focusing more strongly on the factors which make a website with health information a credible source for consumers [35]. Whether Internet health information consumers are able to determine the quality of the information found remains unanswered.

Another line of research assesses quality aspects of health information websites through predefined key word lists evaluating the provided metadata of websites [36]. These measures often combine a checklist for health-relevant words with cross checks of different websites in this domain [37]. Still, these approaches focus on information provided by the hosting provider or institution responsible for the Internet information. Additionally, the provided content is often analyzed for readability and difficult wording [38]. In contrast to these approaches, the aim of this research is to investigate the ability of individuals to distinguish the quality of health information websites. A measure was designed within the context of German language health information on the Internet. The medical condition of sleeping disorders or insomnia was chosen. The procedure for developing and evaluating this measure followed mostly the structured theoretical approach of DeVells [39]. Adaptations were made when combining qualitative and quantitative methods for including the consumer’s perspective, and due to Internet-specific data collection techniques. For the development, observations and structured post observation interviews were used. Based on the findings, a measure was designed. It was tested with a two-group experimental analysis in an Internet survey.

## Methods

### Preliminary Observational Study

To evaluate how a Web search is conducted, 42 naturalistic observations of individuals searching the Internet for information on sleeping disorders were collected. The participants were asked to search for information about sleeping disorders in general; the search was not limited to a distinct perspective or a certain type of sleeping disorder. Following the individual search on the Internet, post observational, structured, in-depth interviews were conducted to clarify users’ motivation for particular search decisions and obtain additional information on their search behavior.

Undergraduate students were instructed to contact volunteer participants in their neighborhood and to observe their searching behavior. The observers were instructed following the guidelines of DeWalt and DeWalt [40]. Particular focus was given to actively observing and taking note of details which would be relevant for the protocols, taking note of possible uncertainties or difficulties of the participants to be clarified in the follow-up interview [40]. Most students contacted the participants in coffee shops where Wi-Fi was available and laptops were being used. To approach them, the student observers were equipped with an observation sheet and interview protocols. The participants received a short study objective beforehand. Participants were informed that this observation was conducted by university students for a research project on health information on the Internet.

The research group designed a field protocol for this study in order to capture the observed setting and contents, following previous recommendations of Schensul et al [41]. The protocols allowed registering the participants’ sociodemographics, the search procedure, the exact search term, their selection from a search results list, the length of time they remained on a website, and the number of results they opened within the observation period. These observation protocols were discussed later in an interview with the participants to collect additional information on their reasons for their choices during the search. In addition, the participants were asked for aspects they remembered from the visited websites. According to Bogdan and Biklin, process codes and activity codes were used to study the participants’ search strategies as described in the protocols [42]. The observation protocols were analyzed following the search procedure of the participants. Similarities and outliers were found by identifying the codes on conferring content equivalence and according to the statements given by the participants.

### Measure Development

Based on the conclusions of the observational study and the interviews, a multi-item measure for the credibility of health websites on the Internet was designed. Orientation for this study was found in the previous work on measures of health information quality assessment [3,7,8,34] and literature reviews in this field [19]. The procedure led to a scale consisting of seven dimensions, each composed of several items, summing up to 49 items in total. The items are designed in the format of statements to which participants can concur or oppose on a seven-point scale ranging from 1 “completely disagree” to 7 “completely agree”. This preliminary scale was critically discussed within the research group, taking the literature into account. Moreover, the single items were checked and pretested with 14 undergraduate students. If necessary, they were adapted, leading to the final measure consisting of seven dimensions and 45 items in total. The dimensions cover several aspects, which were, in the preliminary study, identified as relevant. Among them are more general dimensions (such as layout of the website, textual deficits, usability, and interferences due to advertisement banners and others) and more content-oriented dimensions (such as a trustworthy source, the competence of the authors, and the suitability of the given information for everyday life). The dimensions and numbers of items are presented in Table 1. The items that compose the final measure are shown in Table 4.

**Table 1:** The dimensions of the measure based on the observational study.

Dimension	Number of items	Interest
Trustworthiness	8	Trustworthy source
Competence	7	Content is adequate
Interference	7	Pop-up windows, advertisement
Layout	7	Presentation style
Textual deficits	8	Factor of intelligibility
Usability	4	Access to the information
Suitability	4	Implementation of the advice

### Implementation of the Internet Survey

To test the developed scale, an Internet survey was designed, comparing a group exposed to a low-quality site with another one exposed to a high-quality site. Participants were recruited in two weeks through a snowball system via email, social networks, and online-communities. It was initiated with a sample of 14 undergraduate students. The participants were randomly assigned to one of the two conditions. The high-quality website was rated as such by an independent German consumer foundation involved in investigating and comparing goods and services in an unbiased way [43]. The other website was rated as having low-quality content by the research group in collaboration with sleep experts. For both websites, standardized readability formulas were used to calculate the general reading level. Both websites were of medium complexity. The high-quality website scored 52.61, while the low-quality website scored 47.35 on a scale from 0 (easiest) to 100 (most difficult) [44]. The content of both websites was checked for quality. The key elements were accuracy of the medical information provided, ease of navigation on the website, moderation by the provider, structure and style of content, and if an advertisement could be easily recognized as such. The content of the low-quality website was based on a very general description of insomnia symptoms. Moreover, no sources for the given information were mentioned, which is why it was not clear whether experts or expert knowledge were involved in producing the written content. User comments were neither sorted nor reviewed. Furthermore, it was difficult to distinguish between links for auxiliary insomnia-related content and insomnia-related advertisements. Both were general health websites; only the sections about sleeping disorders were the subject of investigation. For embedding the websites, a HyperText Markup Language (HTML) snippet with the technical restrictions was included into the Internet survey software. HTML is a commonly used markup language for designing Web pages. This Internet survey was administered by a noncommercial and university-based Internet survey platform. Such procedure was inspired by the possibilities offered through digital media and the widely used combinations of research design in offline surveys.

The Internet survey incorporated the websites, and participants had to explore the content for at least four minutes; otherwise it was not possible to continue. The interfaces of the websites were included into the Internet survey mask, while external links on the websites were blocked. Internal paths leading away from sleeping-disorder content were blocked. The quality certificates shown on the high-quality website were removed. The survey was technically pretested before being distributed. After the website exposure, the Internet survey started. The 45 items of the credibility scale and the four items of the outcome measure were presented to each participant in a different random order. At the end of the survey, the participants were asked to respond to questions regarding their Internet usage of health information sites, occupation in a medical profession, and sociodemographic information.

To measure the impact of the website on participants’ behavior, an outcome measure was added. It consisted of four items formulating future intention to consult the site, intention to recommend it, etc (Textbox 1). To achieve a single measure, the items were later averaged. They had the same scaling as the 45 credibility items and were asked in random order together with them.

**The items of the outcome measure as used in the Internet survey.**

Outcome measure:

I would recommend this website

I would approach this source for future questions

I can trust the information on this website

If I suffered from sleeping-disorders, I would use the given information

### Data Analysis

To assess the internal consistency of the measure, a scale reliability analysis was conducted. To check for differences between sociodemographic groups and occupations, respectively, Internet usage for searching medical information, correlations was used. For reasons of sound data analysis, the negatively worded items were reversed using the formula NEWSCORE= (MAX + MIN) – SCORE.

Factors were identified when in the simple structure approach eigenvalues greater than 1.0 were computed [45]. An adequate sample size was checked, using a ratio of five cases to one variable. Following the methodological recommendation presented by Gerbing and Hamilton [46], first a principal component analysis using the Kaiser Normalization and a Varimax rotation was conducted. Moreover a Promax rotation for the identified factors was computed to check their correlations. For the measure of sampling adequacy, factor loadings below .5 were excluded [47]. For all computations, an IBM SPSS Statistics 21 software package was used [48].

## Results

### Observational Study

The participants of the observational study (N=42) were mainly male (25/42, 60%), between 21 and 40 years old, and most had some university degree (20/42, 48%). Table 2 provides a detailed description of these characteristics. The search time was limited to ten minutes by the observers. For the follow-up interviews, between five and ten minutes were needed.

When searching for information on sleeping disorders, all participants used the “Google” search engine as a starting point. Other portals or direct access to websites of medical authorities were not considered. This seems to be in accordance with other recent findings [3,34,49,50]. While some participants were searching for the terms “sleeping disorders” others added a “condition related” term such as “treatment” or “help”. Some participants were very effective in combining these search terms or also using Boolean combinations and sign operators; those that did not had more difficulty finding adequate results, which took more time spent in checking the result list and deciding which website to choose. There were ten participants that exclusively opened results that were displayed on the first result page of the search engine. A page showed a list with ten results; to see more results, participants had to navigate to the next result page. None of the participants checked more than six result pages. Previous research on search behavior notes that the first results are the most likely to be looked at [34]. Sponsored links shown before the results were not taken into consideration in the participants’ search.

In the interviews, the participants were asked individually about their personal observation protocol. They reported that the most relevant key factor for choosing a specific website was its name. The observation protocols showed that a simpler domain name is more likely to be clicked, especially if the search-term was an integral part of the name. As reasons for staying on a website and checking the provided information, most participants mentioned a friendly layout and quality content. Commonly mentioned reasons for leaving were disturbances by advertisement or pop-up boxes and nonadequate information (too general or too specific). About 15 participants stressed the importance of a credible author, such as a governmental institution, a medical association, or professional medical personal, as factors to open or stay on a website.

**Table 2:** Detailed sample description of the observational study, N=42.

Participants		n	%
Total number, N		42	100
**Gender**			
	Male	25	60
	Female	17	41
**Age group, years**			
	17-20	5	12
	21-30	10	24
	31-40	12	29
	41-50	8	19
	50-62	7	17
**Education**			
	No school degree	1	2
	Some school degree	7	17
	High school degree	5	12
	Professional school degree	5	12
	In university education	4	10
	University degree	20	48

### Sample Description of the Internet Survey

The sample of the Internet survey contained 454 participants; 55.1% (250/454) were male, 45.8% (208/454) between 21-30 years, and about 32.2% (146/454) were still at a university. There were 50.2% (228/454) that used the Internet often or very often to search medical information. There were 4.2% (19/454) participants that reported working in the medical sector. In total, the link of the survey was accessed 995 times, implying a completion rate of 45.5% (454/995) among those who had accessed the site. Slightly more of the 454 participants were assigned (51.1%, n=232) to the high-quality website. Analysis of the participants’ Internet protocol (IP) addresses showed that all accessed the survey from a German Internet connection. The IP address is a unique number assigned of the computer used for the survey. A complete sample description is shown in Table 3.

No statistically significant differences could be found between male and female, age groups, Internet usage for health information, and educational levels. Working in the medical sector was negatively related to the ability to distinguish the quality of the website, but due to the small sample size, no further investigation can be done on this point.

**Table 3:** Detailed sample description of the Internet survey.

Participants		Total		Exposure to high-quality page		Exposure to low-quality page	
		n	%	n	%	n	%
Total number, N		454	100	232	100	222	100
**Gender**							
	Male	250	55.1	111	47.8	72	32.4
	Female	171	37.7	99	42.7	139	62.6
	Missing	33	7.3	22	9.5	11	5.0
**Age group, years**							
	15-20	110	24.2	62	26.7	48	21.6
	21-30	208	45.8	90	38.8	118	53.2
	31-40	36	8.0	22	9.5	14	6.3
	41-50	36	8.0	15	6.5	21	9.5
	51-64	29	6.4	20	8.6	9	4.1
	Missing	35	7.7	23	9.9	12	5.4
**Education**							
	No school degree	1	0.2	1	0.4	-	-
	In school education	26	5.7	13	5.6	13	5.9
	Some school degree	59	13.0	37	15.9	22	9.9
	High school degree	82	18.1	44	19.0	38	17.1
	Professional school degree	4	0.9	2	0.9	2	0.9
	In university education	146	32.2	70	30.2	76	34.2
	University degree	135	29.7	64	27.6	71	32.0
	Missing	1	0.2	1	0.4	-	-
**Working in the medical sector**							
	Yes	19	4.2	6	2.6	13	5.9
	No	423	93.2	217	93.5	206	92.8
	Missing	12	2.6	9	3.9	3	1.4
**Internet use for medical information**							
	Not at all	13	2.9	7	3.0	6	2.7
	1 Little	39	8.6	24	10.3	15	6.8
	2	86	18.9	44	19.0	42	18.9
	3	86	18.9	45	19.4	41	18.5
	4	66	14.5	33	14.2	33	14.9
	5	83	18.3	41	17.7	42	18.9
	6	41	9.0	20	8.6	21	9.5
	7 Very often	38	8.4	16	6.9	22	9.9
	Missing	2	0.4	2	0.9	-	-

### Scale Reliability and Principal Component Analysis

By means of the principal component analysis, the different dimensions were tested and the number of items reduced. Out of the 45 items of the scale, four primary factors were identified accounting in total for 65% of overall variance, and following the analysis of the items’ factor loadings and contexts, two factors were recognized as content-specific and the other two as website surrounding-specific factors. The 32 items, which are not part of the final scale, were excluded from further analysis as these displayed high cross-loadings, very low loadings, or no loadings on any factors. Factor 1 accounted for 32.37% (eigenvalue 4.275) of the variance, Factor 2 for 7.96% (eigenvalue 1.035), Factor 3 for 13.37% (eigenvalue 1.738), and Factor 4 for 10.83% (eigenvalue 1.408). The newly grouped items are shown in Table 4.

**Table 4:** Results of the principal component analysis.

	Factors			
	Content-specific^a^		Surrounding-specific^a^	
	1	2	3	4
The content convinced me.	.835			
The website appears to be trustworthy.	.770			
The website provides good information.	.758			
The author seems to be knowledgeable due to the academic title.	.737			
I learned something reading the content.	.688			
The text is too long.		.854		
The sentences have a difficult structure.		.644		
Advertisements distracted me.			.796	
The website contains dispensable links.			.732	
Nothing distracts from the content.			.706	
The website has a blurry layout.			.672	
In general advertisement pop-ups help to add meaningful information.				.853
In general moving advertisement help to draw attention on the content.				.726
Rotation method, Varimax with Kaiser Normalization^b^				

^a^ Extraction method, principal component analysis

^b^ Rotated component matrix; Rotation converged in 5 iterations.

### Factor Labels

Factor 1 was labeled “Trustworthiness” and contained five items on the website being perceived as convincing, trustworthy, and informative (Cronbach alpha=.839). Factor 2 is “Textual deficits” and unites two items on sentence length and complexity (Cronbach alpha=.761). Factor 3, we called “Interference”; it binds items on irritation by advertisements, links, and layout (Cronbach alpha=.592). Finally, Factor 4, “Advertisements”, is on distraction or usefulness of advertisements (Cronbach alpha=.532).

The Promax rotation for four factors showed that there were no correlations higher than the threshold of .32. Following Tabachnick and Fidell [51], we continued with an orthogonal rotation. The results of the oblique rotation are shown in Table 5.

**Table 5:** Factor correlations of the principal component analysis.

Factors^a^	1	2	3
2	.256		
3	-.157	-.218	
4	.052	.067	.198

^a^ Rotation Method, Promax with Kaiser Normalization.

### Differences Between the Conditions

The analysis showed significant differences between the high- and the low-quality websites with regard to the perception of three of the four dimensions, all at a *P*<.001 significance level. Participants who had seen the high-quality website rated it higher on trustworthiness and interference, but lower on textual deficits. Regarding the fourth component, advertisements, both groups rated them as disturbing the users’ focus on content. The difference and the *t*-value were negative, but not significant (*P*=.423). The comparisons can be seen in Table 6. Taken together, these results show that the participants were able to distinguish the quality of medical information on the Internet with regard to trustworthiness and interference, whereas the low-quality site received better ratings on textual deficits. The perception of disturbing advertisements was unrelated to both exposures.

**Table 6:** Statistical differences between the two exposures.

Components	M^a^	SD	df^b^	*t* ^c^	Significance
Trustworthiness (Factor 1)	.778	.112	452	6.970	<.001
Interference (Factor 3)	.821	.134	452	6.132	<.001
Textual deficits (Factor 2)	-.595	.122	452	-4.905	<.001
Advertisements^d^ (Factor 4)	-.107	.134	452	-.802	.423

^a^ M=Mean

^b^ df=degrees of freedom

^c^ *t*=Student’s t distribution

^d^ Equal variances not assumed for this item

### Impact of the Website Quality on the Outcome Measure

The reliability statistics for the four-item outcome measure (see Textbox 1) showed a Cronbach alpha=.853. To find out whether the participants would act differently depending on the quality of the website, an independent sample *t* test was conducted to evaluate the relationship of the outcome measure (Textbox 1) and the content quality. The Levene’s test showed that equal variances could not be assumed. The *t* test showed significant results *t*_446.806_=5.519, *P*<.001. Participants rated the high-quality website (mean 4.46, SD 1.37) in the outcome measure to be better than the website with low-quality content (mean 3.73, SD 1.46). The 95% confidence interval for the difference in means was ranging from 0.47 to 0.99.

##  Discussion

### Principal Findings

This research is based on the experience of average Internet users and quantitative testing of the designed scale. Therefore, it was possible to design a novel measure that covers, on the one hand, similar aspects as the DISCERN scale, but provides, on the other hand, important additional Internet-specific items. The items of the widely used DISCERN measure are divided into two sections that focus on the concepts of quality and credibility of the given information [33]. The items of the presented measure share the importance of constructs measured in the DISCERN, but work differently. In contrast to the existing measure, the items’ structure in the proposed measure is more general and easier for laypersons to assess. It is relevant by taking the particular behavior of Internet information users into consideration. Written information on the Internet can be described as more viral and superficial compared to information found on other sources of mass media, in particular considering the surrounding-specific factors’ interference and advertisements [35,49,50]. The proposed novel measure focuses exclusively on the impressions Internet-users get from the consulted websites. This notion is to date not covered by measures such as DISCERN, but crucial for assessing the credibility impressions of consumers. This proposed measure was developed, therefore, to reflect the behaviors and decisions of individuals searching for health information. In contrast to previously mentioned measures, we did not use samples of individuals with special expertise or professional medical background, but focused exclusively on average Internet-users. Taking together these characteristics, the proposed measure can be combined with existing measures [36-38] on the credibility assessment of health information on the Internet to explore, in a next step, the user perception of the provided health information.

The sufficient level of scale reliability and the properties of this measure suggest that this measure allows examining the view of health information seekers on the provided information. The experimental design showed that the ratings developed for the scale differentiate between a high- and a low-quality website. This makes this measure a useful tool for examining patients’ Internet searches. The measure was not designed based on specific websites, but on the search procedures of the participants of the observational study. Moreover, it is not condition-specific and can be administered to all medical information websites on the Internet. These characteristics allow administering the tool relatively easily in either Internet- or in paper and pencil-based research studies. It can thus be an easy to use measuring tool, which can be incorporated alongside other measures. Useful apps can be found in the eHealth area and for website testing in health campaigns.

Typical for the experimental research layout, several aspects worked differently from what we expected. Between the two experimental groups, the results showed that participants who were exposed to the high-quality website rated its credibility in this measure higher on the factors trustworthiness and interference, but lower on textual deficits. The unexpected direction of the difference could be due to the different styles of the sites. While the high-quality site had long explanatory text parts, the low-quality site had only simple information. Moreover, unexpected results were found on Factor 4 grouping the advertisement items. The nonsignificant results for the correlation of the experimental conditions seem to be reflected within the specific item wording. In contrast to all other items in the final measures, these items could have suggested a more general answer by the participants, which was not limited to the context of the website they had seen. Participants answered this item based on their general attitude and opinion, and consequently, the answers were not affected by the website they had seen. This is reflected by the nonsignificant results of this factor.

Most of the results regarding the rating of the different quality of websites matched with the previous assumption of the research group. For this case, the measure seems to provide a sufficient rating tool able to produce judgments consistent with experts’ categorizations. Although the testing in this study was done on sleeping disorder websites, other conditions can be included. As the measure is by its content not bound to a specific disease or medical condition, it can be widely used. With respect to the growing usage of Internet apps and Internet information by health professionals and laypersons, the measurement catalogue is still very limited when it comes to the combination of content quality and medical information.

### Limitations and Further Research

Initiating a research project with a student sample caused some difficulties overcome by using the snowball system in order to include participants from outside the university. Still, the average age of the sample is rather young and, therefore, does not represent the society of Internet users. It should also be mentioned that health information searches on the Internet are linked to such sociodemographic characteristics as age, gender, and health status [9,10,35]. The presented measure can only be applied to a specific website, but it does not help to understand other relevant determinants such as the result presentation by the search engines. Moreover, the final measure is the result of a statistical analysis, which showed significant effects, but lacks further testing as a composite measure, and, therefore, should be interpreted with caution. This research focused exclusively on one medical condition and did not test the measure with other conditions, which would allow proving the consistency of the measure across different medical subjects. With regard to this aspect, it is unclear how far the measure produces reliable results when considering such controversial medical topics as vaccination or cancer treatment.

Further research with another independent sample will allow confirming the factor structure of the scale. Moreover, it would be possible to provide solutions to some of the limitations and to improve the measure by defining cut-point values as estimators for high- or low-quality content of websites. The measure would in this way offer the possibility of addressing health information users on the Internet who struggle with identifying quality websites. It would also be practical to continue examining this measure in comparison with the health literacy levels of participants to see whether predictors can be found there. So far, the results showed that (formal) knowledge did not show any differences in the research population.

### Conclusions

This measure provides a practical tool, which will show its relevance for research on health information on the Internet. In contrast to previous attempts, this measure is designed for the Internet-setting of this information channel and the particular users’ behavior. The inclusion of the laypersons’ experience into the measurement development process might be seen as unusual, but crucially, this brings the consumers’ perspective into academic research. Therefore, the initially mentioned concern of public health authorities on the quality of health information provided on the Internet [10] can be answered, and the result of this research offers a tool for assessing user perception of content quality. Unlike other information, the impact and the consequences of health information have the potential to be severe. Across gender, age group, and educational level, this measure provides a clear answer on the abilities of participants to estimate the quality of medical information on the Internet. Website testing can be enriched by a credibility criterion based on the ratings of participants. As the amount of medical information on the Internet increases and patients are increasingly empowered to decide on relevant health matters, the research link between general quality assessment and Internet health information becomes relevant. The skill to critically consume health information is important to fully make use of the opportunities and health benefits which eHealth tools offer. From a scientific point of view, the disparities, which can be seen in health literacy levels, will probably be the same when it comes to medical information usage on the Internet. Therefore, understanding how participants perceive medical information on the Internet is a first step to identifying needs and addressing them properly. A measure is ready to be used for the assessment of patients’ perception of credibility of eHealth contents.

## Multimedia Appendix 1

The seven dimensions with the original items of the measure based on the observational study (compare Table 1 in the article) and the original items of the outcome measure (compare Textbox 1 in the article).

## Abbreviations

HTML: HyperText Markup Language
IP: Internet protocol
