# Supplementary material for: Medical Information on the Internet: A Tool for Measuring Consumer Perception of Quality Aspects
Source: Interact J Med Res. 2015 Mar 30;4(1):e8. doi: 10.2196/ijmr.3144 (PMC4395769; doi:10.2196/ijmr.3144)
Supplement: Supplementary file 1 [file ijmr_v4i1e8_app1.pdf]

Appendix 1. The seven dimensions with the original items of the measure based on the observational study (compare Table 1 in the article) and the original items of the outcome measure (compare Textbox 1 in the article). The scale was tested in German language. All items were answered on a seven-point scale ranging from 1 “completely disagree” to 7 “completely agree” and shown in randomized order.

Trustworthiness

01 Die Autoren der Internetseite verfolgen offensichtlich eigennützige Ziele.

02 Die Autoren geben die verwendeten Quellen an.

03 Die Informationen werden neutral dargestellt.

04 Nicht alle Informationen scheinen medizinisch begründet zu sein.

05 Die Auskünfte der Internetseite waren mir bereits bekannt.

06 Man hat den Eindruck überredet zu werden.

07 Die dargestellten Inhalte haben mich überzeugt.

08 Die Internetseite wirkt auf mich vertrauenswürdig.

Competence

09 Die Internetseite informiert gut zum Thema.

10 Man kann annehmen der Autor kennt sich aus, da er einen akademischen Titel trägt (Professor, Doktor, Diplom).

11 Die Informationen der Internetseite sind aktuell (entsprechen den neusten medizinischen Kenntnissen).

12 Meiner Meinung nach handelt es sich um eine unabhängige Internetseite.

13 Ich fand es gut, dass die Internetseite über mehrere Gesundheitsthemen informiert.

14 Ich habe das Gefühl, der Autor weiß nicht wirklich wovon er spricht.

15 Online-Medien sind grundsätzlich nicht für Gesundheitsfragen geeignet.

Interference

16 Die Werbung hat mich gestört.

17 Die Internetseite enthält seriöse Links.

18 Werbe Pop-Ups helfen im Allgemeinen die Informationen auf der Internetseite sinnvoll zu ergänzen.

19 Die Internetseite enthält unwichtige Links.

20 Im Allgemeinen helfen bewegte Werbeanzeigen die Aufmerksamkeit auf das Thema zu lenken.

21 Die Internetseite enthält viele unnütze Informationen.

22 Man ist durch nichts auf dieser Internetseite vom Inhalt abgelenkt.

Layout

23 Die Internetseite ist graphisch ansprechend gestaltet.

24 Die Internetseite wirkt überladen.

25 Das Farbkonzept spricht mich an.

26 Die Internetseite ist klar strukturiert.

27 Die gewählte Schriftart ist angenehm zu lesen.

28 Es ist schlecht erkennbar, was wichtig und was unwichtig ist.

29 Es sind zu viele Bilder im Verhältnis zum Text.

Textual deficits

30 Der Text hat zu viele Fremdwörter.

31 Ich kann den wesentlichen Inhalt Anderen erklären.

32 Den Text kann man auch ohne Vorwissen verstehen.

33 Der Text macht Schwieriges verständlich.

34 Ich muss manche Sätze mehrmals durchlesen um sie zu verstehen.

35 Man hat den Eindruck die Internetseite ist eher für Ärzte gemacht.

36 Die Sätze sind schwer formuliert.

37 Ich habe durch den Text etwas gelernt.

Usabiliy

38 Die gewünschten Informationen sind schwer zu finden.

39 Die Abbildungen verwirren mich.

40 Die Seite ist leicht zu bedienen.

41 Es fällt mir leicht zwischen den Informationen auf der Internetseite hin und her zu springen.

Suitability

42 Die genannten Empfehlungen sind im Alltag einfach umzusetzen.

43 Der Text ist zu lang.

44 Ich kann mit den Informationen nicht viel anfangen.

45 Die Empfehlungen umzusetzen ist mir zu umständlich.

Outcome measure

46 Ich würde diese Internetseite einem Bekannten mit Schlafstörungen empfehlen.

47 Für weitere Fragen würde ich mich wieder an diese Internetseite wenden.

48 Den Informationen auf dieser Internetseite kann ich trauen.

49 Wenn ich unter Schlafstörungen leide, werde ich diese Informationen nutzen.
